# Patient-Specific Simulation of Coronary Artery Pressure Measurements: An *In Vivo* Three-Dimensional Validation Study in Humans

**DOI:** 10.1155/2015/628416

**Published:** 2015-03-01

**Authors:** Panagiotis K. Siogkas, Michail I. Papafaklis, Antonis I. Sakellarios, Kostas A. Stefanou, Christos V. Bourantas, Lambros S. Athanasiou, Themis P. Exarchos, Katerina K. Naka, Lampros K. Michalis, Oberdan Parodi, Dimitrios I. Fotiadis

**Affiliations:** ^1^Unit of Medical Technology and Intelligent Information Systems, Department of Materials Science, University of Ioannina, 45110 Ioannina, Greece; ^2^Department of Cardiology, Medical School, University of Ioannina, 45110 Ioannina, Greece; ^3^Harvard-MIT Division of Health Sciences & Technology, Massachusetts Institute of Technology, Cambridge, MA 02139, USA; ^4^Michailideion Cardiac Center, University of Ioannina, 45110 Ioannina, Greece; ^5^Biomedical Research Institute-FORTH, University of Ioannina, 45110 Ioannina, Greece; ^6^Thoraxcenter, Erasmus Medical Center, 3000 CA Rotterdam, Netherlands; ^7^Istituto di Fisiologia Clinica, Consiglio Nazionale delle Ricerche (IFC-CNR), 56124 Pisa, Italy

## Abstract

Pressure measurements using finite element computations without the need of a wire could be valuable in clinical practice. Our aim was to compare the computed distal coronary pressure values with the measured values using a pressure wire, while testing the effect of different boundary conditions for the simulation. Eight coronary arteries (lumen and outer vessel wall) from six patients were reconstructed in three-dimensional (3D) space using intravascular ultrasound and biplane angiographic images. Pressure values at the distal and proximal end of the vessel and flow velocity values at the distal end were acquired with the use of a combo pressure-flow wire. The 3D lumen and wall models were discretized into finite elements; fluid structure interaction (FSI) and rigid wall simulations were performed for one cardiac cycle both with pulsatile and steady flow in separate simulations. The results showed a high correlation between the measured and the computed coronary pressure values (coefficient of determination [*r* ^2^] ranging between 0.8902 and 0.9961), while the less demanding simulations using steady flow and rigid walls resulted in very small relative error. Our study demonstrates that computational assessment of coronary pressure is feasible and seems to be accurate compared to the wire-based measurements.

## 1. Introduction

Cardiovascular disease is the leading cause of mortality in developed countries. Atherosclerosis develops due to the accumulation of lipids in the arterial wall and the migration of smooth muscle cells to the intima and leukocyte infiltration, thereby forming plaques in the arterial wall. When the progression of atherosclerotic lesions exceeds the compensatory wall response, plaque protrudes into the lumen causing stenosis and obstructs blood flow to the distal myocardial bed.

Lumen obstruction may be hemodynamically significant causing stable angina in patients. One of the most common and efficient ways to assess the hemodynamic significance of coronary lesions is the measurement of the fractional flow reserve (FFR) [1]. FFR is defined as the maximal coronary flow in an arterial segment with a stenosis, divided by the maximal coronary flow in the same arterial segment if no stenosis was present and is measured as the ratio of distal to proximal (i.e., aortic) coronary pressure under maximal vasodilation. Therefore, assessment of coronary pressure is critical for estimating FFR. The measurement of coronary pressure is currently performed invasively with the use of a dedicated pressure wire. However, the advent of technology has now enabled blood flow simulations in three-dimensional (3D) coronary artery reconstructions. Accurate calculation of coronary pressure using finite element simulations without using a pressure wire could be a valuable tool in the catheterization laboratory.

Blood flow simulations are demanding and depend on the applied boundary conditions. A critical boundary condition imposed during the simulation is the behavior of the arterial wall. There are two main approaches for simulating the behavior of the wall. One assumes that the arterial wall is rigid, not taking into consideration the interaction between the blood and the arterial wall [2–6], while the second assumes that arteries are elastic incorporating the interaction between the blood and the arterial walls into the simulation.

The first attempts of blood flow simulations in human arteries were made on 3D simplified tube-like geometries representing arterial segments. Following the advances in image processing, accurate 3D reconstructed arterial models were used for computational blood flow simulations, resulting in more precise results. The rigid wall assumption led to quick blood flow simulations since only the lumen needed to be discretized. However, in an effort to realistically simulate the complexity of the human vasculature, the interaction between the blood and the arterial wall was introduced by applying fluid structure interaction (FSI) models [2, 7–18]. According to these models, blood flow creates loads on the surface of the arterial wall forcing it to deform. The elastic nature of the arterial wall tends to restore the wall to its original state, thereby causing the deformation of the blood domain. Both the blood and the arterial wall domains are discretized and the equations of each domain are solved and used as initial conditions to the other domain. Due to the large number of equations that need to be solved, FSI simulations are very demanding in computational resources and very time-consuming compared to the rigid wall approach but are considered to provide more accurate results for the flow field. However, the differences in computed pressure values between the rigid wall and FSI approaches have not been previously studied.

Currently, we present a validation study for coronary artery pressure measurements using patient-specific 3D coronary artery reconstructions and investigate (a) the accuracy of the computed pressure results using the invasive pressure measurements as the gold standard and (b) the differences in computed pressure measurements between different critical boundary conditions (steady versus pulsatile flow and rigid wall versus FSI).

## 2. Methods

### 2.1. Patient Data

**Figure 1:**
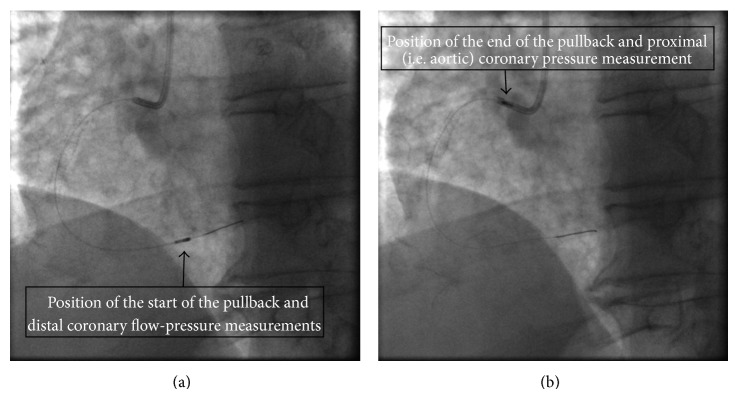
The two views depict the exact locations of the start (a) and end (b) of the pullback procedure as well as the exact positions of the pressure and flow measurements acquisition.

Six subjects underwent intravascular ultrasound (IVUS) and angiography examinations for angina symptoms at CNR (Institute of Clinical Physiology, Milan, Italy). The clinical and demographic patient characteristics are presented in Table 1. A coronary guide wire (0.014 inch diameter) with miniaturized tip transducers for pressure and flow measurements (Combo wire, Volcano Corp.) was used. The pressure-flow wire was inserted in the coronary artery until a stable recording of the flow velocity was obtained at a distal coronary location. The aforementioned parameters were measured at the baseline and during maximal coronary vasodilation (hyperemic conditions) which was achieved with the intravenous administration of adenosine (140 mcg/kg/min). The parameters measured under hyperemic conditions were used as boundary conditions for the simulations and are described in detail in Section 2.3.4. The final measurements included pressure values throughout three cardiac cycles at the proximal (guiding catheter at the ostium of the artery) and distal locations of each arterial segment, combined with flow velocity values at the distal location both at baseline and during maximal hyperemia. A 3-French catheter with a 64-crystal electronic ultrasound probe was used for IVUS examination (Eagle-Eye, Volcano Corp.). The catheter was placed in the distal part of the examined vessel and then a motorized pullback (speed 1 mm/sec) was performed. Following contrast injection two isocentric angiographic views were obtained to depict the position of the catheter inside the vessel before the start of the pullback. The IVUS probe was positioned distally at the same location where the distal coronary pressure-flow measurements were performed so that these measurements could be applied as boundary conditions for the blood flow simulations in 3D reconstructed arterial models as it is described below.  Figure 1 shows the angiographic images with the exact locations of the acquired measurements for the right coronary artery (RCA) of patient 4.

### 2.2. Three-Dimensional Reconstruction

**Figure 2:**
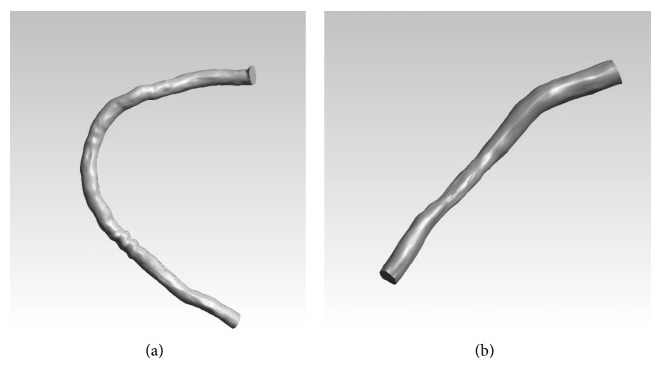
Three-dimensional reconstruction of the lumen of a right coronary artery for patient #2 (a) and patient #1 (b).

The 3D reconstruction of the 8 arterial segments was performed using a methodology which is based on the fusion of IVUS and biplane angiographic data [19]. The end-diastolic frames were selected for segmenting the lumen and the external elastic media (i.e., vessel wall) borders. Then, the corresponding angiographic end-diastolic images were used to reconstruct the 3D IVUS catheter path. The segmented frames were then placed onto the generated 3D catheter path and were appropriately oriented. Finally, two point clouds representing the lumen and vessel wall were derived for each artery and were processed to nonuniform rational B-spline (NURBS) 3D surfaces.  Figure 2 depicts two 3D reconstructed models of two RCA segments. Our dataset includes 4 RCA and 4 LAD segments with mild or moderate lumen stenosis.

### 2.3. Blood Flow Simulation

Transient as well as steady flow simulations were carried out on all 8 arterial segments with either rigid or deformable wall assumptions. In total, four different approaches were used: FSI-transient, FSI-steady flow, rigid walls-transient and rigid walls-steady flow. The most demanding in terms of computational resources is the one using FSI models with transient flow as it is time dependent, whereas the lowest computational requirements are for the one with the rigid walls assumption and the steady flow. The computational approach and the boundary conditions for each type of simulation are presented in detail below.

#### 2.3.1. Rigid Wall Assumption

Blood flow is modeled using the Navier-Stokes and the continuity equations:

(1) $$\begin{array}{c} \rho \frac{\partial v}{\partial t}+\rho \left(v\cdot \nabla \right)v-\nabla \cdot \tau =0,\\ \nabla \cdot \left(\rho v\right)=0, \end{array}$$

where **v** is the blood velocity vector, *ρ* the blood density, and **τ** is the stress tensor, defined as

(2) $$\begin{array}{c} \tau =-p\delta_{ij}+2\mu \varepsilon_{ij}, \end{array}$$

where *δ*_*ij*_ is the Kronecker delta, *μ* is the blood dynamic viscosity, *p* is the blood pressure, and *ε*_*ij*_ is the strain tensor calculated as

(3) $$\begin{array}{c} \varepsilon_{ij}=\frac{1}{2}\left(\nabla v+\nabla v^{T}\right). \end{array}$$

Blood was treated as a Newtonian fluid having a density of 1060 kg/m^3^ and a dynamic viscosity 0.0035 Pa·s. The blood flow was considered laminar with the Reynolds number ranging between 126 and 883.

#### 2.3.2. Fluid Structure Interaction-Blood Domain

In FSI simulations, the interface between the lumen and the wall (i.e., the wall boundary of the fluid domain) deforms, and thus the equations governing fluid flow are expressed in terms of the fluid variables relative to the mesh movement. For the moving reference frame in FSI simulations, the momentum conservation equation for fluid flow is

(4) $$\begin{array}{c} \rho \frac{\partial v}{\partial t}+\rho \left(\left(v-w\right)\cdot \nabla \right)v-\nabla \cdot \tau =0, \end{array}$$

where *ρ* is the density of the blood, **v** is the blood velocity vector, **w** is the vector of the moving mesh velocity (i.e., the velocity of the deformable wall boundary), and **τ** is the stress tensor.

*(a) Fluid Structure Interaction-Arterial Wall Domain.* The following momentum conservation equation is used to model the arterial wall domain:

(5) $$\begin{array}{c} \nabla \tau_{s}+f_{s}^{B}=\rho_{s}{\ddot{d}}_{s}, \end{array}$$

where **τ**_*s*_ is the arterial wall stress tensor, **f**_*s*_^**B**^ are the body forces per unit volume, *ρ*_*s*_ is the density of the arterial wall, and ${\ddot{d}}_{s}$ is the solid's local acceleration.

Due to lack of universal values for the parameters of the material properties of the arterial wall, we have used a nine-parameter Mooney-Rivlin model to describe the material properties of the wall. Despite the fact that the coronary arterial wall is considered to have an anisotropic and heterogeneous structure due to the complex composition (e.g., collagen fibers), we applied an isotropic and homogenous material model because of the absence of* in vivo* data regarding the fiber direction and the heterogeneity that describes the anisotropic behavior of the arterial tissue. The parameters of the Mooney-Rivlin model were set as previously described in FSI analyses in the human right coronary artery [18, 20]. The following equation is used to calculate the strain energy function:

(6) W=c10I−1−3+c01I−2−3+c20I−1−32+c11I−1−3I−2−3+c02I−2−32+c30I−1−33+c21I−1−32I−2−3+c12I−1−3I−2−32+c03I−2−33+1dJ−12.

${\overset{-}{I}}_{1}$, ${\overset{-}{I}}_{2}$ are the first and second deviatoric strain invariants, respectively, and *J* is the determinant of the elastic deformation gradient tensor. The rest of the parameters are set as in [18]: *c*_10_ = 0.07 MPa, *c*_20_ = 3.2 MPa, and *c*_21_ = 0.0716 MPa and the others are equal to zero. The compressibility parameter *d* is defined as

(7) $$\begin{array}{c} d=\frac{2}{K}, \end{array}$$

where *K* is the bulk modulus (1 × 10^−5^).

#### 2.3.3. Fluid Structure Interaction-Coupling Equations

In order for the two domains to be solved together, the following displacement compatibility and traction equilibrium equations must be satisfied:

(8) $$\begin{array}{c} \tau_{s}\cdot {\hat{n}}_{s}=\tau_{f}\cdot {\hat{n}}_{f}\;\;\left(x,y,z\right)\in \Gamma_{\text{FSI}}^{S}\cap \Gamma_{\text{FSI}}^{F}, \end{array}$$

(9) $$\begin{array}{c} d_{s}=d_{f}\;\;\left(x,y,z\right)\in \Gamma_{\text{FSI}}^{S}\cap \Gamma_{\text{FSI}}^{F}, \end{array}$$

where Γ_FSI_^*S*^ is a set of points on the arterial wall and Γ_FSI_^*F*^ a set of points on the lumen.

The generated stresses from the fluid and the solid on the interface of the two domains must be in equilibrium (8) and the displacements of the two domains on their common surface must be equal (9).

#### 2.3.4. Boundary Conditions

**Figure 3:**
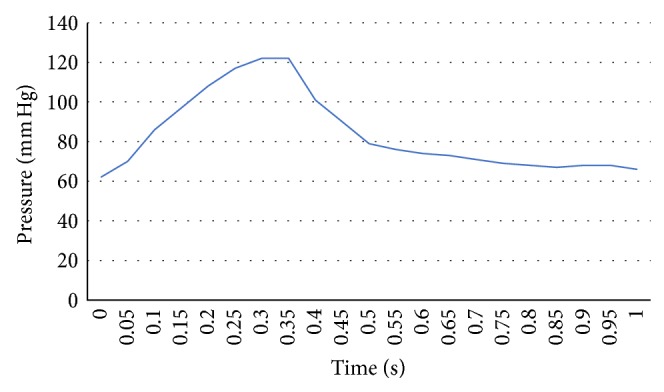
Measured pressure profile for patient #6 for a full cardiac cycle.

*(i) Inlet*. Regarding the inlet, a measured pressure profile in the catheterization laboratory was applied as a boundary condition. In particular, for the transient simulations, a full cardiac cycle (either the second or the third measured in order for the measurements to be stable and accurate) was divided into time steps of 0.05 seconds (Figure 3 exhibits the applied inlet pressure profile for patient 6), while for the steady flow simulations, the mean pressure value of the same cardiac cycle that was used in the transient ones was applied as the inlet boundary condition.

**Figure 4:**
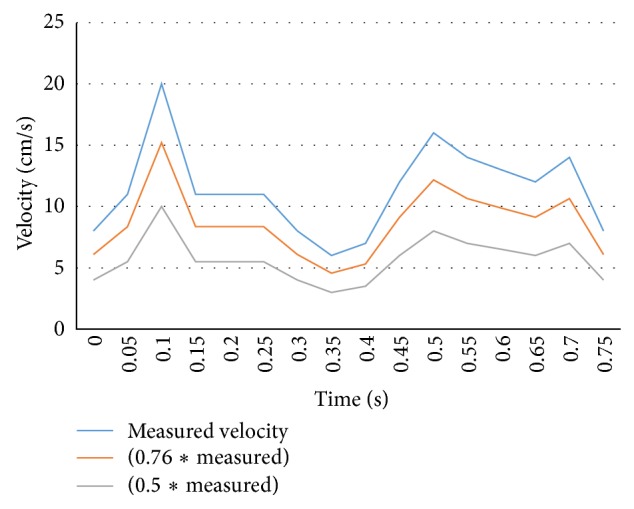
Mean velocity values calculated for patient #1 in order to determine the optimal velocity profile for validation.

*(ii) Outlet*. Velocity profiles were available at the distal end of the reconstructed artery (measured invasively using the combo pressure-flow wire) and were prescribed as outlet boundary conditions. To capture the true nature of the velocity profile of the outlet, we used the developed flow (this has a paraboloid profile) derived from the 3D geometry and we defined the “magnitude” of the developed flow according to the flow measurements. To achieve that, we applied the mass flow rate profile for each case which was calculated as

(10) $$\begin{array}{c} \dot{m}=\rho vA, \end{array}$$

where *ρ* is the blood's density, **v** is the velocity of blood, and *A* is the cross-sectional area of the outlet. However, due to the nature of the Doppler wire measurements, we executed a parametric study regarding the accuracy of the measured velocity values. The measured velocity values from the wire cannot be considered to be the highest of the cross section due to the fact that either the wire is not aligned in the center of the vessel or due to the fact that the wire itself interrupts the flow. The velocity value that is inserted in the mass flow rate equation is the mean velocity value of the profile. We tried three different velocity profiles to examine which fits our problem best. In the first case scenario, the measured values from the Doppler wire as the mean profile value were used; in the second scenario a ratio of 0.76 (**v**_mean_ = 0.76∗**v**_measured_) as it was previously suggested [21]; and in the third scenario a ratio of 0.5 which is common in the generalized Poiseuille flow.  Figure 4 depicts the velocity profiles of the three cases for an RCA segment of patient 1. The closest results to the measured values were achieved by using the measured velocity values as the mean value of the profile. The results of the parametric study are presented in detail in Section 3.

*(iii) Lumen Wall Interface*. At the lumen wall, a no-slip boundary condition was applied, meaning that the blood had zero velocity relative to the solid-fluid interface.

*(iv) Arterial Wall.* The distal ends of the arterial wall (inlet and outlet) were assumed to be fixed on all directions so that motion was restricted at these sites.

#### 2.3.5. Mesh

The lumen was discretized into hexahedral elements, with an element face size ranging from 0.09 to 0.12 mm, with an increased mesh density throughout the boundary layer of the flow close to the arterial wall. The arterial wall was discretized into tetrahedral elements with an element face size 0.09 mm and 15 layers of brick elements with a thickness of 0.03 mm at the interface with the lumen. The brick element layers were first generated from the interface of the wall and the lumen towards the outer perimeter of the wall and then the remaining volume was discretized into tetrahedral elements.

The mesh size both for the lumen and the wall was selected after performing a mesh (face size) sensitivity analysis. The sensitivity analysis was performed in a representative case both for the rigid (Table 4) and deformable (Table 5) wall assumption using steady-state flow. The mesh sensitivity analysis for the deformable wall simulation (Table 5) was performed using a face size of 0.09–0.12 mm for the lumen (as derived from the initial sensitivity analysis for the rigid wall assumption in Table 4). The analysis was based on the correlation between the mesh size and the produced results regarding the average wall shear stress of the same cross-section on 4 different mesh sizes. The mesh size with <5% difference in wall shear stress values was used in the final simulations; of note, computed pressure values at the outlet, on which we focus in the current study, were also minimally influenced by the mesh size (<0.05% difference, Tables 4 and 5).

## 3. Results

A series of blood flow simulations using different assumptions and approaches was carried out, a linear regression analysis on all 8 vessels was performed, and the respective aggregate Bland-Altman plot was obtained in order to examine the correlation of the computed results to the measured ones.

### 3.1. Validation Results

**Figure 5:**
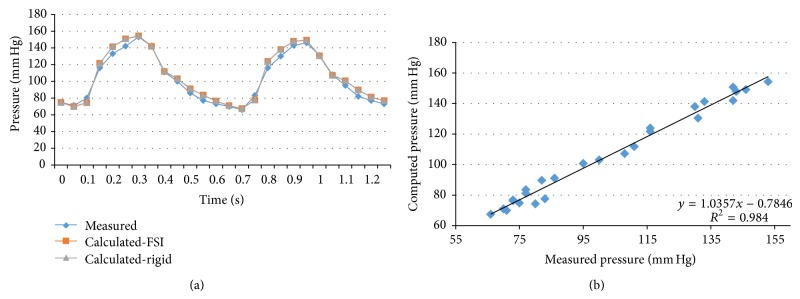
(a) depicts the pressure waveforms for the examined cardiac cycles (measured and calculated results) and (b) exhibits the linear regression analysis for patient #1.

**Figure 6:**
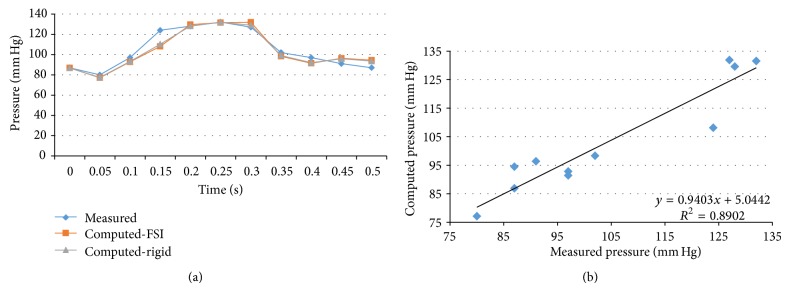
(a) depicts the pressure waveforms for the examined cardiac cycles (measured and computed results) and (b) exhibits the linear regression analysis for patient #2.

**Figure 7:**
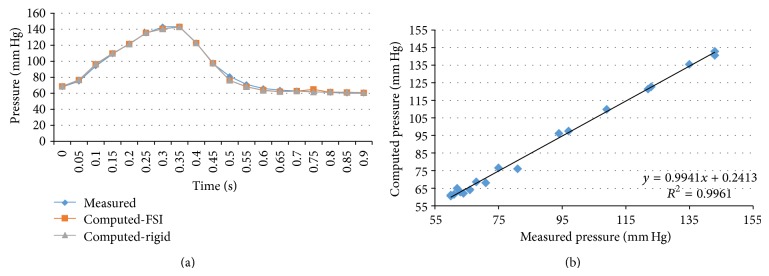
(a) depicts the pressure waveforms for the examined cardiac cycles (measured and computed results) and (b) exhibits the linear regression analysis for patient #3.

**Figure 8:**
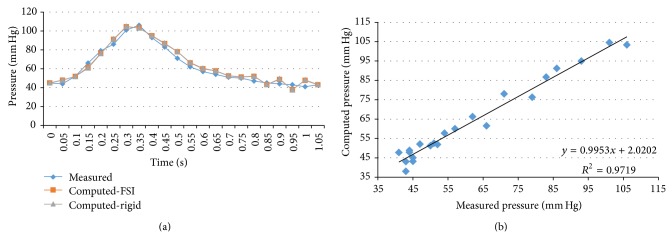
(a) depicts the pressure waveforms for the examined cardiac cycles (measured and computed results) and (b) exhibits the linear regression analysis for patient #4, right coronary artery.

**Figure 9:**
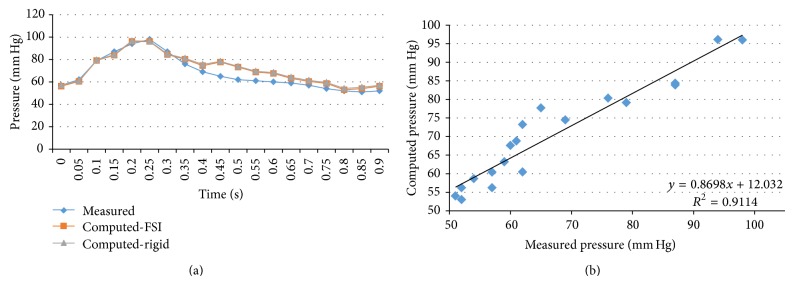
(a) depicts the pressure waveforms for the examined cardiac cycles (measured and computed results) and (b) exhibits the linear regression analysis for patient #4, left anterior descending coronary artery.

**Figure 10:**
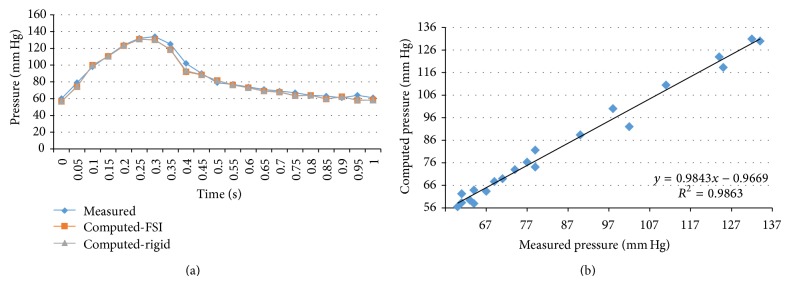
(a) depicts the pressure waveforms for the examined cardiac cycles (measured and computed results) and (b) exhibits the linear regression analysis for patient #5, right coronary artery.

**Figure 11:**
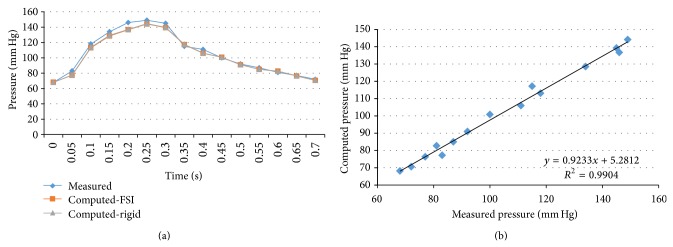
(a) depicts the pressure waveforms for the examined cardiac cycles (measured and computed results) and (b) exhibits the linear regression analysis for patient #5, left anterior descending coronary artery.

**Figure 12:**
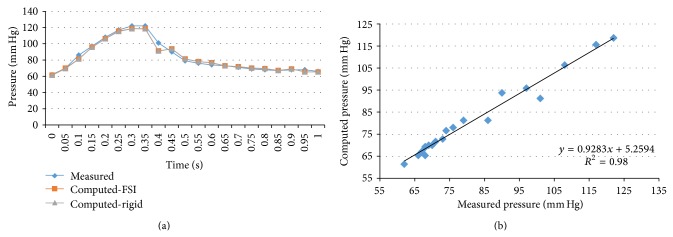
(a) depicts the pressure waveforms for the examined cardiac cycles (measured and computed results) and (b) exhibits the linear regression analysis for patient #6.

**Figure 13:**
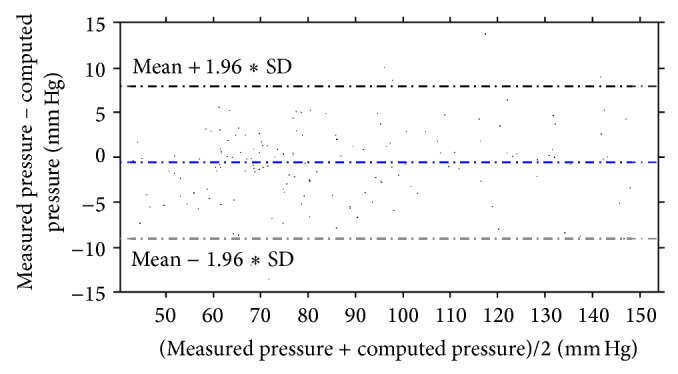
Bland-Altman plot for all 8 cases.

We performed transient FSI simulations for one cardiac cycle. The produced results show excellent correlation between the measured and the calculated values with the worst case scenario having a coefficient of determination *r* ^2^ = 0.8902 and the best case scenario having an *r* ^2^ = 0.9961. The Bland-Altman plots also depict a high similarity between the measured and the computed values with almost all values being within the 1.96∗SD cut-offs. Figures 5, 6, 7, 8, 9, 10, 11, and 12 depict the pressure waveforms of the measured and the rigid wall computed as well as the FSI computed values and the linear regression analysis plots for all cases. Moreover, Figure 13 represents an aggregate Bland-Altman plot for all 8 cases with a mean difference close to zero.

### 3.2. Rigid Wall versus FSI Simulations

The calculated mean difference between the rigid wall and the FSI simulations for all cases reached the statistically negligible value of 0.26%. The rigid wall simulations produced slightly higher pressure values than the FSI simulations on most of the examined cases. Moreover, compared to the values measured in the catheterization laboratory, and the FSI simulations produced slightly more accurate results than the rigid wall ones. In Table 2, a comparison between the measured and the computed mean outlet pressure values for all cases is presented.

### 3.3. Transient versus Steady Flow Simulations

The computed pressure of the steady flow simulation was compared to the average pressure of the same cardiac cycle as it was computed from the transient simulation. Our results demonstrated a very close match between the steady flow and the transient results for both rigid and FSI simulations. In detail, the two simulation types exhibited a mean difference of 0.44% (Table 2). The results that were closest to the measured wire-based values were the ones obtained using the transient simulations as expected.  Table 3 demonstrates the results of the parametric study related to the flow velocity values used in the mass flow rate equation for the outlet boundary condition. It seems that the optimal results were obtained when the measured flow velocity values from the combo wire were used as the maximum and not the mean values of the velocity profile.

## 4. Discussion

We presented a study on coronary artery pressure measurements using blood flow simulation in realistic 3D reconstructed coronary arteries. Our primary findings are the following: (I) computed distal coronary pressure values correlate very well with the measured ones using the pressure wire and (II) the assumption of rigid walls and steady flow results in negligible differences compared to the more demanding FSI and pulsatile simulations, respectively.

Several validation studies have been previously carried out to test the accuracy and validity of numerical methods. Phantom, simplified 3D models, or patient-specific arterial models have been previously employed in order to perform blood flow simulations. Left coronary artery bifurcations and carotid bifurcations, as well as mesenteric arterial segments were included. The computed velocity profiles were then compared to the measured ones resulting in a fairly good agreement between the measured and the computed values [22–27]. A recent study examined the correlation of flow and pressure patterns between the computed and the measured values for two deformable flow phantoms mimicking a normal and an obstructed aorta, respectively [28]. Good qualitative agreement was found between the measured and the computed values for flow, exhibiting a better correlation for the pressure results. The majority of those studies focus on the carotid vasculature due to the technical difficulty that arises when dealing with the coronary vasculature. Coronary arteries require invasive imaging methods in order to acquire information related to the size and complex anatomy of the obstruction. Therefore, there is a lack of data on the accuracy of the results regarding numerical simulations in human coronary arteries.

In our study, we focus on coronary arteries and use realistic patient-specific reconstructed coronary arteries derived from angiographic and IVUS data. Furthermore, we use* in vivo* data from invasive flow/pressure measurements in the catheterization laboratory for our validation purposes. The results exhibited a very high correlation of the computed pressure values compared to the measured ones. The pressure waveforms between the measured and the computed values distally in coronary arteries were very close to each other, and the mean computed pressure values for each case showed very small relative error values. Moreover, there was a very good agreement between the measured and the computed values. In addition, our findings demonstrate that the less demanding simulations using steady flow and rigid walls instead of pulsatile flow and FSI result in very small relative error. Therefore, our results support the use of the simpler and less time-consuming simulations for coronary artery pressure computation.

*Clinical Implications and Challenges.* Hemodynamic factors such as arterial pressure both proximal and distal to coronary stenoses are of great clinical importance. FFR, calculated as the ratio of distal to proximal coronary pressure under maximal vasodilation, has been shown to discriminate functionally significant stenoses and help in patient management leading to favorable clinical outcomes [29]. Our results support the use of numerical simulations for assessing distal coronary pressure in humans. This approach implemented in 3D realistic human coronary arteries could open the pathway to FFR assessment based on imaging data only without the need of a pressure wire. However, several challenges lie in the pathway of virtual FFR assessment including the “*a priori*” selection of the appropriate boundary condition for hyperemic flow, the incorporation of the resistance of the distal myocardial bed into the simulation, and the effect of flow division in the branched coronary tree. Although our results demonstrated that finite element simulation in realistic 3D coronary models may yield accurate distal pressure measurements if aortic pressure and coronary flow are known, further clinical studies are needed to test the accuracy of virtual pressure measurements when patient-specific hemodynamic conditions at the inlet are not known.

*Limitations.* The reconstructed segments in the current study neglect the presence of bifurcations which influence flow distribution. Moreover, the hemodynamic significance (i.e., pressure drop) of the coronary stenoses in the arteries studied was not large, and thus we did not have the opportunity to test the accuracy of the computed pressure values in cases with large pressure gradients.

## 5. Conclusions

Our study highlights the value of numerical simulations applied in 3D models for assessing hemodynamic factors such as coronary artery pressure. The accuracy of the computed results supports the use of this approach for virtual pressure calculation which may have major clinical implications for assessing the hemodynamic significance of coronary stenoses without using a pressure wire in the catheterization laboratory.

## Figures and Tables

**Table 1:** Patient demographic and clinical characteristics.

Patient	Age	Sex	Familiarity	Hypertension	Hypercholesterolemia	Diabetes	Angina
01	73	M	N	Y	Y	Y	N
02	55	M	N	Y	Y	N	Y
03	56	M	Y	Y	Y	Y	N
04	56	M	N	Y	Y	Y	N
05	70	M	Y	Y	Y	N	N
06	75	M	N	Y	Y	N	N

**Table 2:** Comparison between the wire-based measured pressure values (*P*_out_) and the computed values from the four types of simulations (*P*_out(comp)_).

Patient #	*P*_out_ (mm Hg)	FSI-transient *P*_out(comp)_ (mm Hg)	Rigid-transient *P*_out(comp)_ (mm Hg)	FSI-steady state *P*_out(comp)_ (mm Hg)	Rigid-steady state *P*_out(comp)_ (mm Hg)
1-RCA	103.04	105.93	106.06	105.46	105.48
2-RCA	104.73	103.52	103.38	103.82	103.71
3-LAD	89.32	89.03	89	89.17	89.15
4-RCA	61.95	63.68	63.83	64.01	64.22
4-LAD	67.47	70.72	71.45	70.94	71.59
5-RCA	105.2	102.42	102.68	102.51	102.58
5-LAD	85.95	83.64	83.85	83.59	83.72
6-LAD	83.52	82.8	82.77	82.98	83.03

**Table 3:** Results of the parametric study concerning the velocity profiles (steady-state simulations).

Patient #	*P*_out(comp)_ (mm Hg)	*P*_out_(*v*_max⁡_) (mm Hg)	*P*_out_(0.76 ∗ *v*_max⁡_) (mm Hg)	*P*_out_(0.5 ∗ *v*_max⁡_) (mm Hg)
1-RCA	103.04	105.48	106.16	106.75
2-RCA	104.73	103.71	106.56	107.6
3-LAD	89.32	89.15	90.48	91.19
4-RCA	61.95	64.22	65.02	65.66
4-LAD	67.47	71.59	73.21	74.68
5-RCA	105.2	102.58	103.18	103.53
5-LAD	85.95	83.72	84.07	84.55
6-LAD	83.52	83.03	85.23	86.96

**Table 4:** Results of the mesh sensitivity analysis in the lumen (rigid wall assumption).

Face size	Lumen mesh size (elements)	Outlet pressure (mm Hg)	Difference in pressure (%)	Cross-sectional WSS (Pa)	Difference in WSS (%)
0.13–0.15 mm	87 K	105.516	0.053	6.31	21.22
0.12–0.15 mm	176 K	105.512	0.049	7.14	10.86
**0.09**–**0.12 mm**	**400 K**	**105.483**	**0.022**	**7.78**	**2.87**
0.07–0.09 mm	657 K	105.460	—	8.01	—

WSS: wall shear stress.

The selected mesh size for the final simulations is indicated in bold font (<5% difference in WSS).

**Table 5:** Results of the sensitivity analysis in the deformable wall assumption (lumen face size was 0.09–0.12 mm).

Element size	Wall mesh size (elements)	Outlet pressure (mm Hg)	Difference in pressure (%)	Cross-sectional WSS (Pa)	Difference in WSS (%)
0.13 mm	292 K	105.494	0.048	6.38	21.62
0.10 mm	540 K	105.487	0.042	7.19	11.67
**0.09 mm**	**582 K**	**105.458**	**0.014**	**7.85**	**3.56**
0.07 mm	1.232 M	105.443	—	8.14	—

WSS: wall shear stress.

The selected mesh size for the final simulations is indicated in bold font (<5% difference in WSS).
